# Complementarity determining regions in SARS-CoV-2 hybrid immunity

**DOI:** 10.3389/fimmu.2023.1050037

**Published:** 2023-02-21

**Authors:** Glynis Frans, Doreen Dillaerts, Tom Dehaemers, Jan Van Elslande, Jonas De Leeuw, Lise Boon, Wim Maes, Nico Callewaert, Bas Calcoen, Lina Ancheva, Maaike Cockx, Nick Geukens, Kusay Arat, Rita Derua, Pieter Vermeersch, Sebastien Christian Carpentier, Xavier Bossuyt

**Affiliations:** ^1^ Department of Laboratory Medicine, University Hospitals Leuven, Leuven, Belgium; ^2^ Clinical and Diagnostic Immunology, Department of Microbiology, Immunology and Transplantation, KU Leuven, Leuven, Belgium; ^3^ PharmAbs: The KU Leuven Antibody Center, KU Leuven, Leuven, Belgium; ^4^ Clinical Laboratory, AZ Groeninge Hospital, Kortrijk, Belgium; ^5^ Laboratory for Thrombosis Research, KU Leuven Kulak Kortrijk, Kortrijk, Belgium; ^6^ SyBioMa, KU Leuven, Leuven, Belgium; ^7^ Department of Molecular and Cellular Medicine, Laboratory of Protein Phosphorylation and Proteomics, KU Leuven, Leuven, Belgium; ^8^ Department of Cardiovascular Sciences, KU Leuven, Leuven, Belgium

**Keywords:** SARS-CoV-2, COVID-19, hybrid immunity, complementary determining region, vaccination

## Abstract

Pre-vaccination SARS-CoV-2 infection can boost protection elicited by COVID-19 vaccination and post-vaccination breakthrough SARS-CoV-2 infection can boost existing immunity conferred by COVID-19 vaccination. Such ‘hybrid immunity’ is effective against SARS-CoV-2 variants. In order to understand ‘hybrid immunity’ at the molecular level we studied the complementarity determining regions (CDR) of anti-RBD (receptor binding domain) antibodies isolated from individuals with ‘hybrid immunity’ as well as from ‘naive’ (not SARS-CoV-2 infected) vaccinated individuals. CDR analysis was done by liquid chromatography/mass spectrometry-mass spectrometry. Principal component analysis and partial least square differential analysis showed that COVID-19 vaccinated people share CDR profiles and that pre-vaccination SARS-CoV-2 infection or breakthrough infection further shape the CDR profile, with a CDR profile in hybrid immunity that clustered away from the CDR profile in vaccinated people without infection. Thus, our results show a CDR profile in hybrid immunity that is distinct from the vaccination-induced CDR profile.

## Introduction

People who recovered from previous SARS-CoV-2 infection mount a stronger and more rapid response to COVID-19 vaccination with an increased durability than people who had not been previously infected (1). Moreover, serum of ‘previously infected’ vaccinated people is better able to neutralize immune-evading strains than serum of ‘naive’ vaccinated people who had never been infected with SARS-CoV-2 (2). Vaccination enhanced and prolonged protection (against symptomatic infection, severe outcome, hospitalization) of previously infected people (3–5).

Immunity induced by prime/boost vaccination declines over time and a third vaccination efficiently restores immunity against Alpha, Beta, Gamma and Delta variants of the virus, but less so against the Omicron variant (6). Analysis of memory B cell-derived antibodies elicited after vaccination or infection confirmed that Omicron evaded neutralization by a large proportion of these antibodies (7). Boosting previously vaccinated nonhuman primates with a mRNA-Omicron variant elicited similar levels of protection compared to boosting with a non-Omicron-specific vaccine (8). Conversely, Suryawanshi et al. showed that SARS-CoV-2 Omicron breakthrough infection induced higher neutralization titers against variants of concern than SARS-CoV-2 Omicron infection in unvaccinated individuals (9). Relatedly, Kahn et al. showed that Omicron (breakthrough) infection enhanced Delta antibody immunity in vaccinated persons (10). Thus, breakthrough SARS-CoV-2 infections enrich antibody immunity in vaccinated people.

Taken together, natural infection boosts the magnitude and quality of the humoral immune response to vaccination, irrespective of whether the infection occurs before or after vaccination (11). Such enhanced immune protection in individuals who have had one or more doses of a COVID-19 vaccine and experienced a SARS-CoV-2 infection before or after the vaccination is defined as ‘hybrid immunity’ (WHO). However, it remained unclear whether the enhanced protection conferred by hybrid immunity is solely related to increased levels of the neutralizing antibodies or also to a more diverse, mature antibody repertoire. In order to evaluate at the molecular level whether ‘hybrid immunity’ differs from immunity induced in ‘naive’ (uninfected) vaccinated people, we interrogated the antibody repertoire by analysis of the variable complementary determining regions (CDRs) of spike protein receptor-binding domain (RBD)-specific antibodies using liquid chromatography/tandem mass spectrometry (LC/MS-MS).

## Methods

### Ethical approval

The study was approved by the ethics committee of the University Hospitals Leuven and AZ Groeninge Hospital Kortrijk and participants signed informed consent (S64152, S64089, S2021005 [AZG Kortrijk]). The individuals included in this study have been included in previous studies (12, 13).

### Reagents

Purified SARS-CoV-2 spike antigen (S1-RBD) [His-Tag (HEK293)] was purchased from the Native Antigen Company [REC31882-500].

Tris-HCl was from Invitrogen. Dithiothreitol (DTT), iodoacetamide (IAM) and urea were from Sigma-Aldrich, digestion enzymes endoproteinase Lys-C and chymotrypsin from Thermo Scientific and MS grade formic acid and acetonitrile from Biosolve.

### IgG anti-S antibody measurement

IgG antibodies against SARS-CoV-2 spike RBD were measured with the Abbott Architect (Abbott, Lake Forest, Illinois) SARS-CoV-2 IgG II Quant chemiluminescence immunoassay using the manufacturer’s cut-off for positivity of 50 AU/mL. Values exceeding 4.160 AU/mL (“high titer”) have been proposed as a surrogate for the presence of high neutralizing antibody titer (12).

### Neutralizing antibody measurement

Neutralizing antibodies were measured by SARS-CoV-2 NeutraLISA from Euroimmun (Lübeck, Germany). In this assay, neutralizing antibodies compete with biotinylated ACE2 receptors to attach to recombinant S1 antigen coated onto the microplate wells. Data are expressed as % inhibition: negative: <20, borderline ≥20 to <35, positive ≥35.

### RBD-specific B lymphocytes

RBD-specific B lymphocytes were quantitated as described by Calcoen et al. (13).

### Anti-RBD enrichment

Costar plates were coated with RBD (0,5 µg per well) and left overnight at 4°C. The following day, coated plates were washed thrice with PBS-T (0,05% Tween-20 in PBS). Diluted serum (1/10 in PBS-T) was added and the plates were incubated in a thermomixer (RT, 800 rpm, 1 h). After incubation, plates were washed three times with PBS-T, followed by washing twice in PBS. Finally, plates were washed with 50 mM Tris-HCl.

It should be noted that this enrichment procedure not only enriches IgG antibodies but also IgM and IgA class antibodies. This was substantiated by MS-based demonstration of immunoglobulin heavy constant mu-specific (prototypic) peptides (e.g. AATSQVLLPSK, AIPPSFASIF) in addition to heavy constant gamma-specific peptides in all experiments (data not shown). Immunoglobulin heavy constant alpha 1 was present in some but not all cohorts.

### Digestion with Lys-C/Chymotrypsin

Following enrichment, 60 µl 4 M urea/50 mM Tris-HCl was added to each well. Afterwards, reduction of proteins was executed for 1h in the thermomixer (800 rpm, 37°C) in the presence of 8 mM DTT, followed by alkylation for 30 min in the dark (37°C) in the presence of 22 mM IAM. Digestion was started by addition of Lys-C at 0,4 µg/well and left overnight (thermomixer 37°C, 800 rpm, dark). The next day, 50 mM Tris-HCl was added until urea concentration dropped below 1 M. Chymotrypsin was added at 1 µg/well and digestion of the proteins continued in the thermomixer (4 h, 37°C, 800 rpm). Finally, digestion was stopped in the presence of 1% FA.

### Desalting

Desalting was performed on Sep-Pak 96-well tC18 µElution plates using a vacuum pump. To activate the column, 200 µl 50% acetonitrile (ACN) was added to each well and the pump was activated for 1 min. This activation step was repeated once. During equilibration, 200 µl 0.5% formic acid (FA)/5% ACN was added to each well and the pump was activated for 1 min. This equilibration step was repeated once. Next, samples were loaded on the column and the pump was activated. The flow-through was passed through the column again to ensure maximum binding. Afterwards, the column was washed 4 times with 0.5% FA/5% ACN. To elute samples, 50 µl 70% ACN was loaded on the column, the pump was activated and the flow-through was collected in a new 96-well plate. Samples were transferred to Eppendorf 1.5 ml tubes and dried in the Speedvac centrifuge (Uniequip Univapo 150 ECH). Dried samples were stored at -20°C.

### MS analysis + preparation

Dried samples were resuspended in 15 µl 0.1% FA/5% ACN before being injected (5 μL) and separated on an Ultimate 3000 UPLC system (Dionex, Thermo Scientific) equipped with an Acclaim PepMap 100 pre-column (C18 3 μm–100 Å, Thermo Scientific) and a C18 PepMap RSLC (2 μm, 50 μm-15 cm, Thermo Scientific) using a linear gradient (300 nL/min) of 0–4% buffer B (80% ACN, 0.08% FA) in 3 min, 4–10% B in 7 min, 10–35% in 25 min, 35–38% in 5 min, 38–40% in 2 min, 40–65% in 5 min, 65–95% in 1 min, 95% for 9 min, 95–5% in 1 min, 5% for 9 min.

The Q Exactive Orbitrap mass spectrometer (Thermo Scientific) was operated in positive ion mode using data-dependent acquisition with a survey MS scan at a resolution of 70,000 (FWHM at m/z 200), followed by MS/MS scans (resolution 17,500) of the top ten most intense peaks with +2, +3, +4, and +5 charged ions above a threshold ion count of 16,000 using normalized collision energy (NCE) of 25 eV with an isolation window of 2.0 m/z, apex trigger of 5-15 s and dynamic exclusion of 30 s. All data were acquired with Xcalibur 3.1.66.10 software (Thermo Scientific).

Measurements were taken from distinct samples.

MS data are available *via* ProteomeXchange with identifier PXD038849.

### Bio-informatics analysis

Progenesis software (version 4.1; Nonlineair Dynamics Ltd, New Castle, UK) was used for relative quantification of data. To correct for possible variation, the samples were normalized based on the commonly detected abundance values (median and mean absolute deviation outlier filtering approach, “normalize to all proteins” option in Nonlineair Dynamics Ltd, New Castle, UK). Mascot (version 2.2.06; Matrix Science Inc., London, UK) was used for identification of peptides by searching against the Uniprot *Homo sapiens* database (194,319 entries).

Peptides with a Mascot peptide sequence score ≥20 and identified as immunoglobulin (Ig)-like were further aligned to databases containing V, D or J region germline sequences.

Peptides with a Mascot score ≥20 identified as non-Ig-like or with a Mascot score <20 were analyzed by *de novo* sequencing. *De novo* sequencing was performed starting from the raw data files using the PEAKS Studio software (Version 10.6; Bioinformatics Solutions Inc., Waterloo, Canada). A *de novo* score (Average of Local Confidence, ALC%) was assigned by PEAKS based on the reliability of each amino acid in a *de novo* sequence. Only *de novo* peptides with scores of ALC% >80% were included for further analysis (VDJ alignment).

Included peptide sequences were subsequently aligned to databases containing V, D or J-region germline sequences derived from IMGT database (14) using IgBLAST (15) and IMGT/DomainGapAlign (16) algorithms. Peptides with sufficient match (IgBLAST criteria for FR and CDR1/2: E-value ≤ 0.05; IMGT criteria for CDR3: E-value ≤ 1, Smith-Watermann score >30, and min. 3 amino acids aligned to CDR3) to the human immunoglobulin V-region databases were assigned to a frame region (FR) or complementarity determining region (CDR) of the corresponding immunoglobulin germline gene. Only peptides that were assigned to a CDR (with or without amino acids overlapping neighboring FRs) were used for further statistical analysis. An overview of the bio-informatics analysis workflow is given in **Extended Data Figure 1**.

### Statistical analysis

Sparse Partial Least Squares Discriminant Analysis (sPLS-DA) of the normalized abundance values of the identified V-region immunoglobulin CDR peptides was performed to identify the most predictive/discriminative CDR peptides to classify infection/vaccination groups (17, 18). Three steps were performed for each experiment in the sPLS-DA analysis. First, sPLS-DA models require a X and Y matrix as input. For each experiment, the normalized abundance values per CDR peptide for each patient was used as the X matrix and the group of each patient was used as the Y matrix. Second, two sPLS-DA parameters were tuned based on the Balanced Error Rate and maximum distance metric: the optimal number of components (H) and the optimal number of variables (= peptides) to select on each component. Third and final, sPLS-DA models were calculated for H components (max. 5) with the component-specific optimal number of peptides using 3-fold cross-validation with 50 repeats. Peptides that were included in the resulting sPLS-DA model were labeled as “discriminative peptides” for the corresponding experiment. sPLS-DA was performed using the MixOmics package (version 6.20.0, ref 1) in R (version 4.20.0).

The discriminative CDR peptides that were induced by vaccination/infection for each experiment were used as input for PCA (principal component analysis) and visualized in PCA and heat maps using Qlucore Omics Explorer (QIucore, Lund, Sweden).

## Results

We evaluated the CDR profile of RBD-specific antibodies before and after COVID-19 vaccination in naive and previously infected people, as well as in vaccinated people with a breakthrough infection. Vaccination was performed with two doses of BNT162b2 (second dose after 3 weeks) in January-April 2021. The prevailing SARS-CoV-2 variants were wild-type for the individuals that were infected before vaccination and alpha or delta for the individuals that suffered from a breakthrough infection. The RBD-specific antibodies were enriched using RBD-coated ELISA plates, digested into peptides and finally analyzed by LC-MS/MS (see Methods). Peptides sequences were obtained through database search by Mascot using the Uniprot *Homo sapiens* database and through *de novo* sequencing using PEAKS Studio software (see Methods). Peptide sequences were confirmed as immunoglobulin-derived CDR regions by alignment with the IMGT database using IgBLAST and IMGT/DomainGapAlign software (see Methods).

### COVID-19 vaccination-induced CDR profile in naive and previously infected individuals

**Figure 1:**
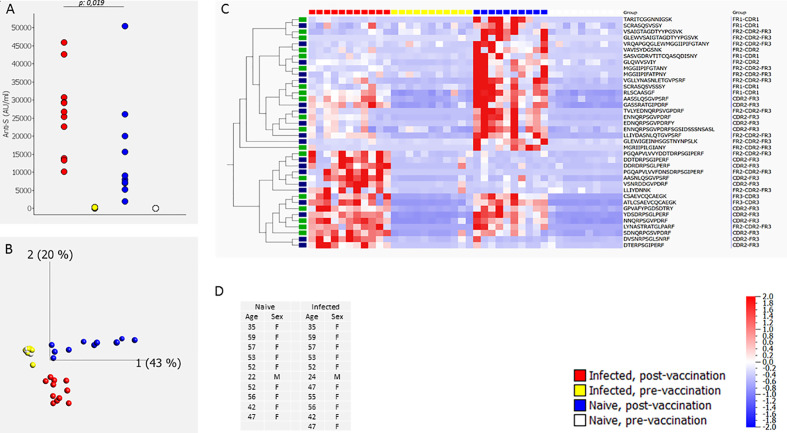
CDR profile and IgG anti-S antibodies before and after two doses of the BN162b2 vaccine [6 weeks (40-45 days) after first dose, three weeks after the second dose] in naïve individuals and in individuals that had been infected with SARS-CoV-2 before vaccination (determined by a positive SARS-CoV-2 PCR on a respiratory sample or presence of IgG anti-S and/or IgG anti-N in serum before infection) (experiment #1). The figure shows the anti-S antibody levels **(A)**, the PCA analysis **(B)**, heat map after hierarchical clustering of the CDR features **(C)** and age and gender distribution of the included individuals **(D)**. The heat map indicates the variance (from -2 to 2) compared to the mean (0) for each peptide feature. The included vaccination/infection-induced CDR-derived peptides were those that revealed discriminative by sPLS-DA analysis. The green-labeled CDR-derived peptides were also revealed discriminative by sPLS-DA analysis in experiment #2 (**Extended Data Figure 2**). The median [25^th^-75^th^ percentile] anti-S antibody levels (AU/mL) were 2 [1.8-3.3], 8483 [7028-18901], 122 [86-243], and 26771 [18235-30021] pre-vaccination naïve, post-vaccination naïve, pre-vaccination infected and post-vaccination infected, respectively.

In a first set of experiments we compared the post-vaccination CDR profile in individuals that had been infected with SARS-CoV-2 before vaccination to the CDR profile in individuals that had not been previously infected (naive). The CDR profile (and anti-S antibody level) was determined before and 6 weeks after vaccination (two doses of the BN162b2 vaccine). A first experiment (#1) included 10 naive and 11 infected individuals (**Figure 1D**) and a second experiment (#2) 9 naive and 9 infected individuals (**Extended Data Figure 2D**). Discriminative CDR peptides were determined by sparse Partial Least Squares Discriminant Analysis (sPLS-DA) of the identified CDR peptides in each experiment. sPLS-DA identified 39 and 59 discriminative peptides in experiment #1 and experiment #2, respectively, 38 of which were induced by vaccination/infection in experiment #1 and 56 in experiment #2, respectively. Nineteen peptides were commonly found in both experiments. **Figure 1** shows, the principal component analysis (PCA) (Panel B) and heatmap (Panel C) of the sPLS-DA output, as well as the anti-S antibody levels for experiment #1 (Panel A). The results for experiment #2 are shown in **Extended Data Figure 2**. The post-vaccination anti-S antibody levels in the infected individuals were significantly different (higher) from the antibody levels in the naive individuals (p=0.019 for experiment #1, p=0.0008 for experiment #2; Mann-Whitney-U) (**Figure 1A**). The post-vaccination neutralizing capacity was high (~100% inhibition) in the naïve and previously infected individuals (**Extended Data Table 1**). PCA analysis of both experiments shows that the post-vaccination CDR profile of the infected individuals could be clearly separated from the profile of the naive individuals, whereas the pre-vaccination profiles coincided (**Figure 1B** and **Extended Data Figure 2B**). The heat maps revealed different subsets of peptides (**Figure 1C** and **Extended Data Figure 2C**). Some peptides were only induced in naive people, whereas others were induced by vaccination in infected as well as in naive people. A small set of peptides was mainly found in people with hybrid immunity. A heat map representing only the nineteen common peptides found in experiment 1 and 2 is shown in **Extended Data Figure 3**.

### COVID-19 vaccination-induced CDR profile and breakthrough infection

**Figure 2:**
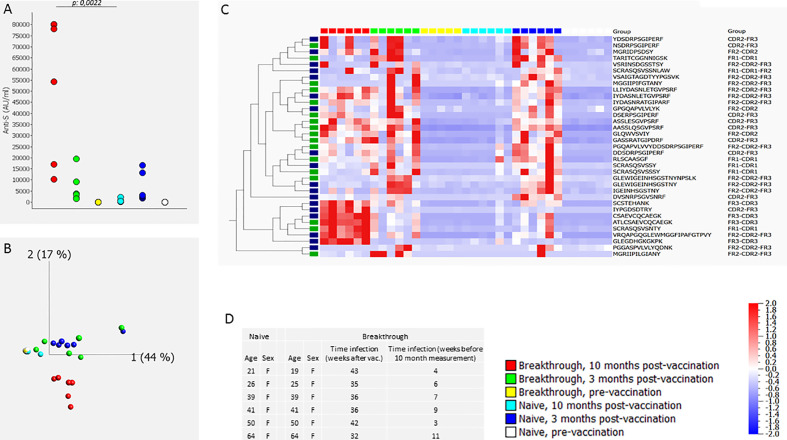
CDR profile and anti-S antibodies before, 3 months and 10 months after BN162b2 vaccination (two doses) in naive individuals and in individuals that experienced a breakthrough infection between 3 and 10 months after vaccination (experiment #3). The figure shows the anti-S antibody levels **(A)**, the PCA analysis **(B)**, heat map after hierarchical clustering of the CDR features **(C)** and age and gender distribution with timing of the breakthrough infection of the included individuals **(D)**. The heat map indicates the variance (from -2 to 2) compared to the mean (0) for each peptide feature. The included vaccination/infection-induced CDR-derived peptides were those that revealed discriminative by sPLS-DA analysis. The green-labeled CDR-derived peptides were also revealed discriminative by sPLS-DA analysis in experiment #4 (**Extended Data Figure 4**). The median [25^th^-75^th^ percentile] anti-S antibody levels (AU/mL) were 2 [0.2-3.47], 2914 [2453-10717] and 374 [278-531], 2 [0.5-2.7], 3621 [2152-7819], 66209 [26454-79519], pre-vaccination no breakthrough, post-vaccination 3 months no breakthrough, post-vaccination no breakthrough, pre-vaccination breakthrough, post-vaccination 3 months breakthrough, post-vaccination breakthrough, respectively.

In a third experiment (#3) we compared the CDR profile in 6 individuals vaccinated with BN162b2 who were SARS-CoV-2 naive before vaccination but experienced a breakthrough infection between 3 and 10 months after vaccination to the CDR profile in 6 BN162b2 vaccinated SARS-CoV-2 naive individuals (no breakthrough infection). The CDR profile was determined before vaccination, 3 months after vaccination and 10 months after vaccination. Results are shown in **Figure 2**. Ten months after vaccination, anti-S antibody levels in individuals with a breakthrough infection were significantly different (higher) (p=0.0022: Mann-Whitney-U) from the antibody levels in individuals without breakthrough infection (**Figure 2A**). Similarly, neutralizing activity 10 months after vaccination was higher in individuals that experienced a breakthrough infection between 3 months and 10 months after vaccination (> 97.8% inhibition) than in individuals that did not experience a breakthrough infection (neutralizing activity varying between 10% and 90% inhibition) (**Extended Data Table 1**). sPLS-DA identified 35 discriminatory peptides of which 35 were induced by vaccination/infection. PCA analysis of the sPLS-DA output revealed that in naïve people the CDR profile at 3 months was separated from the pre-vaccination CDR profile (**Figure 2C**). The CDR profile at 10 months was comparable to the pre-vaccination CDR profile in individuals without breakthrough infection, but clearly clustered away from any other condition in individuals with a breakthrough infection (**Figure 2B**). The heatmap of these peptides revealed vaccination-specific peptides (3 months after infection) that were further induced upon natural infection, as well as vaccination-specific CDR peptides that were not further induced by natural infection (**Figure 2C**). A second comparable but smaller experiment (#4) including 2 individuals with and 3 without breakthrough infection revealed a similar outcome (**Extended Data Figure 4**). In this experiment, sPLS-DA revealed 45 discriminatory peptides, of which 44 were induced by vaccination/infection and of which 17 overlapped with experiment #3. For experiment 4, data on RBD-specific B lymphocytes was available (13). RBD-specific B lymphocytes evaluated 10 months after vaccination amounted to 0.30% and 0.25% of total living B lymphocytes in two individuals with a breakthrough infection and 0%, 0% and 0.02% in 3 individuals without a breakthrough infection, indicating that the breakthrough infection expanded RBD-specific memory B lymphocytes.

There were 13 peptides that overlapped between experiment #1 (infection before vaccination) and experiment #3 (infection after vaccination) (**Extended Data Figure 5**). Remarkably, hybrid-specific peptides (i.e. found in vaccinated and infected people) [e.g. AASSLQSGVPSRF and (ATL)CSAEVCQCAEGK] overlapped between the two experiments, as did peptides that were vaccination-specific but not further induced by infection [e.g. VSAIGTAGDTYYPGSVK]. For all experiments, the discriminative peptides, the region, the gene and whether they were detected *via de novo* sequencing is given in **Extended Data Table 2**. For genes with non-discriminative allele calls, the allele with the highest prevalence was derived from a North European population using VDJbase (19, 20).

## Discussion

Our data substantiated that the RBD-specific antibody levels generated by vaccination were higher in previously infected individuals than in naïve individuals and that breakthrough infections induced an increase in antibody titers and neutralizing capacity. Our results further indicated a convergent CDR profile upon vaccination. Vaccinated people shared CDR sequences as determined in serum by LC-MS/MS. Pre-vaccination infection or breakthrough infection further shaped the CDR profile, with a CDR profile in hybrid immunity that clustered away from the CDR profile in vaccinated individuals without infection. Thus, our results show a CDR profile in hybrid immunity (irrespective of whether the infection is before or after vaccination) that is distinct from the vaccination-induced CDR profile and, therefore, provide supporting evidence for altered (enhanced) immunity in individuals with hybrid immunity.

Our findings of a convergent CDR profile are in line with previous observations that SARS-CoV-2 specific memory B cells from (naïve and COVID-19 disease-recovered) vaccinated individuals harbored a high frequency of convergent RBD-specific clones (21).

Even though it is unknown to what extent the CDR profile in serum reflects the CDR repertoire in memory B cells, our findings in serum are in keeping with the observations of Sokal et al. (21) who described that maturation and acquirement of somatic mutations after vaccination was more pronounced in SARS-CoV-2 recovered individuals than in naïve individuals (21). Sokal et al. (22) also showed that COVID-19 infection induced an immediate response, including pre-existing cross-reactive seasonal Beta-coronavirus-specific clones, and an ongoing antigen-driven activation with accumulation of somatic mutations in the variable region of the memory B cells over time. These somatically mutated memory B cells should give rise to neutralizing antibody secreting cells upon reinfection (22).

An MS-based approach is a powerful tool to document the CDR repertoire of secreted antibodies. It has, however, several limitations. The approach is less sensitive than next generation sequencing to document the memory B cell receptor repertoire, which was exemplified by the fact that we found only few CDR3-related peptides. Somatic hypermutation, as a result of antigen-driven diversification, might remain undetectable by MS as the many variants will each have a low concentration. Thus there might be a tendency to detect less mutated variants (e.g. in IgM class antibodies). We observed more light chain sequences than IGHV sequences, which might be related to more extensive diversity (and lower abundance) of the IGHV sequences. Moreover, CDR analysis by MS in serum is unable to link light and heavy chains of distinct antibodies. It should be noted that analysis at the univariate level (post-vaccination versus pre-vaccination) revealed more vaccination-induced CDR sequences than those revealed by s PLS-DA analysis of all conditions.

The use of data-independent MS analysis, future advances in sensitivity of MS instrumentation, and advances in *de novo* sequencing techniques might improve CDR profiling in serum.

In conclusion, applying an MS approach to evaluate CDRs of serum antibodies revealed that the CDR profile in hybrid immunity is distinct from the vaccination-induced CDR profile.

## Data availability statement

The mass spectrometry proteomics data presented in this study are deposited to the ProteomeXchange Consortium via the PRIDE partner repository with the dataset identifier PXD038849 and 10.6019/PXD038849 (23).

## Ethics statement

The studies involving human participants were reviewed and approved by Ethics committee University Hospitals Leuven and AZ Groeninge Hospital Kortrijk. The patients/participants provided their written informed consent to participate in this study.

## Author contributions

XB conceptualized the study. LA, SC, LB, GF, NG, RD, and KA created and applied the methodology for the study. WM, BC, NC, JV, and PV collected samples. GF, DD, TD, JDL, RD, and XB did the data analysis and verified the data. XB, MC, and NG were involved in funding acquisition. GF was involved in bioinformatics, and RD, GF, TD, and XB in visualization. XB, GF, and TD wrote the original draft of the manuscript. All authors reviewed and edited the manuscript. XB, GF, DD, and TD had access to the raw data. All authors contributed to the article and approved the submitted version.
