# Supplementary material for: Complementarity determining regions in SARS-CoV-2 hybrid immunity
Source: Front Immunol. 2023 Feb 21;14:1050037. doi: 10.3389/fimmu.2023.1050037 (PMC9990870; doi:10.3389/fimmu.2023.1050037)
Supplement: Supplementary file 1 [file DataSheet_1.pdf]

Supplementary Information

Legends to Figures Extended Data

Extended Data Figure 1

Overview of the bio-informatics analysis to process the raw LC-MS/MS data into CDR peptide sequences. LS-MS/MS output and peptide sequences fulfilling the criteria specified in the boxes with grey letters were excluded for further analysis. Software packages for data pre-processing and statistical analyses are specified in the blue boxes. Identified CDR peptides (indicated in bold) were used for further statistical analysis: MixOmics (R) and Qlucore Omics Explorer. ALC: Average of Local Confidence.

Extended Data Figure 2

CDR profile and anti-S antibodies before and after two doses of the BN162b2 vaccine (6 weeks after first dose) in naïve individuals and in individuals that had been infected with SARS-CoV-2 before vaccination (experiment #2).

The figure shows anti-S antibody levels (Panel A), PCA analysis (Panel B), heat map after hierarchical clustering of the CDR features (Panel C) and age and gender distribution of the included individuals (Panel D). The heat map indicates the variance (from -2 to 2) compared to the mean (0) for each peptide feature. The included vaccination/infection-induced CDR-derived peptides were those that revealed discriminative by sPLS-DA analysis. The green-labeled CDR-derived peptides were also revealed discriminative by sPLS-DA analysis in experiment #1 (Figure 1).

The median [25th-75th percentile] anti-S antibody levels (AU/mL) were 5 [1.5-5.6], 8698 [7182-13332], 873 [459-2462], and 36834 [35238-41898] pre-vaccination naïve, post-vaccination naïve, pre-vaccination infected and post-vaccination infected, respectively.

Extended Data Figure 3

Heat maps representing 19 CDR-derived peptides that revealed discriminative by sPLS-DA in both experiment #1 and experiment #2. Heat maps are shown for experiment #1 and #2, as well as for experiment #3 and #4 as far as the CDR-derived peptides were discriminative by sPLS-DA in experiments #3 and/or #4. The heat maps indicate the variance (from -2 to 2) compared to the mean (0) for each peptide feature.

Extended Data Figure 4

CDR profile and anti-S antibodies before, 3 months and 10 months after BN162b2 vaccination (two doses) in naive individuals and in individuals that experienced a breakthrough infection between 3 months and 10 months after vaccination (experiment #4).

The figure shows the anti-S antibody levels (Panel A), PCA analysis (Panel B), heat map after hierarchical clustering of the CDR features (Panel C) and age and gender distribution of the included individuals with timing of the breakthrough infection (Panel D). The heat map indicates the variance (from -2 to 2) compared to the mean (0) for each peptide feature. The included vaccination/infection-induced CDR-derived peptides were those that revealed discriminative by sPLS-DA analysis. The green-labeled CDR-derived peptides were also revealed discriminative by sPLS-DA analysis in experiment #3 (Figure 2).

Extended Data Figure 5

Heat maps representing 13 CDR-derived peptides that revealed discriminative by sPLS-DA in both experiment #1 and experiment #3. Heat maps are shown for experiment #1 and #3, as well as for experiment #2 and #4 as far as the CDR-derived peptides were discriminative by sPLS-DA in experiments #2 and/or #4. The heat maps indicate the variance (from -2 to 2) compared to the mean (0) for each peptide feature.

Extended Data Table 1. Neutralizing activity

Experiment 1 - Neutralizing activity (% Inhibition)			
Naive		Infected	
Pre-vaccination	Post-vaccination	Pre-vaccination	Post-vaccination
-3,5	99,6	-8,2	101,9
1,1	98,7	-13,6	99,3
-9,7	100,2	-4,4	99,6
-15,4	95,9	4,9	98,8
-14,0	68,8	0,8	101,8
-3,5	98,8	-8,6	98,6
-7,6	99,2	1,6	99,5
-10,3	82,0		99,5
-7,6	97,4	15,8	104,8
-1,9	98,5	8,0	102,7
		0,5	97,7

Experiment 2 - Neutralizing activity (% Inhibition)			
Naive		Infected	
Pre-vaccination	Post-vaccination	Pre-vaccination	Post-vaccination
-7,5	95,2	16,9	100,7
-6,6	97,9	24,3	107,9
-8,6	103,5	46,5	104,4
-12,5	97,5	22,0	99,8
-3,7	102,8		100,4
-7,5	40,1	70,4	110,2
0,1	98,7	103,0	107,2
-14,5	103,8	56,0	100,4
-11,6	99,6	40,5	101,5

Experiment 3 - Neutralizing activity (% Inhibition)

Naive			Breakthrough infection (between 3 and 10 months post-vaccination)		
Pre-vaccination	3 months post-vaccination	10 months post vaccination	Pre-vaccination	3 months post-vaccination	10 months post vaccination
-10,5	100,8	12,7	-1,5	101,1	115,2
-6,5	110,0	16,5	-3,4	75,4	100,1
-3,7	102,8	10,1	0,8	102,1	97,8
-1,7	100,3	26,0	6,9	97,0	111,8
11,6	102,3	49,9	-3,5	80,7	101,5
-5,0	105,2	90,2		102,2	98,3

Experiment 4 - Neutralizing activity (% inhibition)

Naive			Breakthrough infection (between 3 and 10 months post-vaccination)		
Pre-vaccination	3 months post-vaccination	10 months post vaccination	Pre-vaccination	3 months post-vaccination	10 months post vaccination
-1,2	90,7	44,7	-18,7	87,6	98,0
-14,2	83,9	22,9	-16,6	84,9	97,0
-9,9	93,0	67,3			

Extended Data Table 2.

Overview of discriminatory peptides found by sPLS-DA analysis for all four experiments, with immunoglobulin region and gene names. For peptides identified by *de novo* sequencing, the PEAKS *de novo* score is indicated. For peptides identified in the Uniprot *Homo sapiens* database, the Mascot score is indicated.

Allele calls were derived from the IMGT database and contain only functional immunoglobulin genes. ORFs and pseudogenes were excluded. For most regions, the peptide was not discriminative for a specific immunoglobulin allele using an IgBLAST query. For genes with non-discriminative allele calls, the allele with the highest prevalence was identified using data from a North European study on 49 healthy controls and 50 celiac patients (European Nucleotide Archive Project PRJEB26509) in VDJbase (1,2). The most prevalent alleles are indicated in bold. Alleles which are not present in VDJbase are indicated with "not available" (NA) and were not used to determine the most prevalent allele.

1. Omer A, Shemesh O, Peres A, et al. VDJbase: an adaptive immune receptor genotype and haplotype database. *Nucleic Acids Res*. 2020; 48: D1051–D1056. doi: 10.1093/nar/gkz872.

2. Gidoni, M., Snir, O., Peres, A. et al. Mosaic deletion patterns of the human antibody heavy chain gene locus shown by Bayesian haplotyping. *Nat Commun*. 2019; 10: 628 . doi: 10.1038/s41467-019-08489-3.

Peptides induced by vaccination/infection – *De novo* sequencing

					PLS-DA Discriminatory peptides				PEAKS *de novo* Score (%)			
Region	Subject_ID	Allelic frequency observed in (2)	Sequence	*De novo*	Exp. 1	Exp. 2	Exp. 3	Exp. 4	Exp. 1	Exp. 2	Exp. 3	Exp. 4
FR3-CDR3	**IGKV1-17*01**	93/99	CLLNNFYPREAK	✓				✓	\	90	\	89
	IGKV1-17*02	0/99										
	IGKV1-17*03	37/99										
	**IGKV1D-17*01**	96/99										
CDR2-FR3	**IGLV3-21*02**	67/99	DDRDRPSGLPERF	✓	✓	✓			86	83	\	\
	IGLV3-21*03	27/99										
CDR2-FR3	**IGLV3-27*07**	NA	DSERPSGLPERF	✓		✓			88	88	\	\
CDR2-FR3	IGLV2-14*03	40/99	DVSNRPSGGKDRF	✓				✓	\	\	\	88
	**IGLV2-14*04**	61/99										
	IGLV2-14*05	NA										
	IGLV2-18*01	57/99										
	**IGLV2-18*02**	96/99										
	IGLV2-18*03	0/99										

CDR2-FR3	**IGLV2-14*04**	61/99	DVSNRPSGLSNRF	✓	✓				94	\	\	\
CDR2-FR3	**IGLV2-14*04**	61/99	DVSNRPSGVSNRF	✓		✓	✓		95	95	97	\
CDR2-FR3	IGLV6-57*01	43/99	EDNQRPSGVPDRF	✓				✓	\	\	\	84
	**IGLV6-57*02**	68/99										
	IGLV6-57*03	25/99										
	IGLV6-57*04	NA										
CDR2-FR3	IGLV6-57*01	43/99	EDNQRPSGVPDRFY	✓	✓				96	96	\	\
	**IGLV6-57*02**	68/99										
	IGLV6-57*03	25/99										
	IGLV6-57*04	NA										
CDR2-FR3	**IGLV2-14*01**	64/99	EVSNRPSGVSSRF	✓		✓			96	96	\	\
FR3-CDR3	**IGLV9-49*01**	94/99	GLEGDHGKGKPK	✓			✓		\	\	82	\
	IGLV9-49*02	1/99										
	IGLV9-49*03	0/99										
CDR2-FR3	**IGLV1-50*01**	10/99	GNSNRPSGVPDYRAGSK	✓		✓			84	84	\	\
FR2-CDR2	**IGLV3-27*01**	98/99	GPGQAPVLVLYK	✓		✓	✓		94	94	87	96
CDR2-FR3	**IGHV5-51*01**	99/99	GPVAFYPGDSDTRY	✓	✓	✓			80	80	\	\
	IGHV5-51*02	0/99										
	IGHV5-51*03	42/99										
	IGHV5-51*04	0/99										
	IGHV5-51*05	0/99										
	IGHV5-51*06	0/99										
	IGHV5-51*07	0/99										
FR2-CDR2-FR3	**IGKV3-15*01**	98/99	LYNASTRATGLPARF	✓	✓	✓			84	84	\	\
	**IGKV3D-15*01**	99/99										
	IGKV3D-15*02	0/99										
CDR2-FR3	**IGLV10-54*02**	27/99	NDRPSGLSDRF	✓		✓			92	92	\	\
	IGLV10-54*04	NA										
CDR2-FR3	**IGLV1-40*01**	99/99	NGTNRPSGVPDRF	✓		✓			80	80	\	\
	IGLV1-40*02	0/99										

	IGLV1-40*03	0/99										
	**IGLV2-14*05**	NA										
	IGLV2-18*01	57/99										
	**IGLV2-18*02**	96/99										
	IGLV2-18*03	0/99										
CDR2-FR3	**IGLV1-44*01**	99/99	NNNQRPSGVPDRF	✓				✓	\	84	\	80
CDR2-FR3	**IGLV1-44*01**	99/99	NNQRPSGVPDRF	✓	✓	✓			92	92	\	\
	IGLV1-44*02	NA										
	**IGLV1-47*01**	94/99										
	IGLV1-47*02	22/99										
	IGLV1-47*03	0/99										
CDR2-FR3	**IGHV3-9*01**	78/99	NSNSLNYADSVK	✓		✓			96	96	\	\
	IGHV3-9*02	0/99										
	IGHV3-9*03	2/99										
	IGHV3-9*04	NA										
FR2-CDR2-FR3	**IGLV3-1*01**	99/99	PGGASPVLVLYQDNK	✓			✓		\	\	87	\
FR2-CDR2-FR3	**IGLV3-21*02**	67/99	PGGQAPVLVVYDDPVKPFVEGDSW	✓		✓			84	84	\	\
	IGLV3-21*03	27/99										
FR2-CDR2-FR3	**IGHV5-51*01**	99/99	PGVLMHPGDSDTRY	✓		✓			81	81	\	\
	IGHV5-51*02	0/99										
	IGHV5-51*03	42/99										
	IGHV5-51*04	0/99										
	IGHV5-51*05	0/99										
	IGHV5-51*06	0/99										
	IGHV5-51*07	0/99										
CDR3	**IGKV3D-7*01**	62/99	QDYLLPEAK	✓		✓			81	81	\	89
FR3-CDR3	**IGLV5-52*01**	89/99	SCSTEHANK	✓			✓		\	\	84	\
CDR2-FR3	IGLV6-57*01	43/99	SDNQRPGSVPDRF	✓	✓	✓			87	87	\	\
	**IGLV6-57*02**	68/99										
	IGLV6-57*03	25/99										

	IGLV6-57*04	NA										
CDR2-FR3	**IGHV3-9*01**	78/99	SLGNFKTTPEDTMGNLGYADSVK	✓		✓			80	80	\	\
	IGHV3-9*02	0/99										
	IGHV3-9*03	2/99										
	IGHV3-9*04	NA										
CDR1-FR2	**IGHV2-26*01**	99/99	SLSNARMGVSW	✓		✓			\	85	89	\
	IGHV2-26*02	0/99										
	IGHV2-26*03	0/99										
	IGHV2-26*04	1/99										
FR2-CDR2-FR3	IGLV6-57*01	43/99	TVLYEDNQRPSVGPDRF	✓	✓				84	\	\	\
	**IGLV6-57*02**	68/99										
	IGLV6-57*03	25/99										
	IGLV6-57*04	NA										
FR2-CDR2-FR3	**IGKV1-33*01**	NA	VGLLYNASNLETGVPSRF	✓	✓				89	\	\	\
	**IGKV1D-33*01**	NA										
FR2-CDR2-FR3	**IGHV3-13*01**	77/99	VSALGTAGDTYYSGPVK	✓				✓	\	\	\	90
	IGHV3-13*03	0/99										
	IGHV3-13*04	22/99										
CDR2-FR3	**IGKV2-30*01**	97/99	VSNRDDGVPDRF	✓	✓				89	89	\	\
	IGKV2-30*02	13/99										
CDR2-FR3	IGLV3-21*01	0/99	YDSDRPSGLPERF	✓	✓			✓	98	\	\	96
	**IGLV3-21*04**	56/99										

Peptides induced by vaccination/infection – Uniprot *Homo sapiens* database

					PLS-DA Discriminatory peptides				MASCOT score			
Region	Subject_ID	Allelic frequency observed in (2)	Sequence	*De novo*	Exp. 1	Exp. 2	Exp. 3	Exp. 4	Exp. 1	Exp. 2	Exp. 3	Exp. 4
CDR2-FR3	**IGKV1-37*01**	NA	AASNLQSGVPSRF		✓	✓			48,2	49,8	48,1	\
	**IGKV1D-37*01**	99/99										
CDR2-FR3	**IGKV1-12*01**	NA	AASSLQSGVPSRF		✓	✓	✓	✓	74,3	74,2	61,7	69,9
	**IGKV1-12*02**	NA										

	**IGKV1-16*01**	1/99									
	**IGKV1-17*01**	93/99									
	IGKV1-17*02	0/99									
	IGKV1-17*03	37/99									
	**IGKV1-39*01**	NA									
	**IGKV1-6*01**	96/99									
	IGKV1-6*02	0/99									
	**IGKV1D-12*01**	NA									
	**IGKV1D-12*02**	NA									
	**IGKV1D-16*01**	97/99									
	IGKV1D-16*02	18/99									
	**IGKV1D-17*01**	96/99									
	**IGKV1D-39*01**	NA									
CDR2-FR3	**IGLV1-44*01**	99/99	ANNNRPSGVPDRF		✓			22,7	29,0	\	\
	IGLV1-44*02	NA									
	**IGLV1-47*01**	94/99									
CDR2-FR3	**IGLV1-40*01**	99/99	ANSNRPSGVPDRF		✓			15,6	20,5	\	\
	IGLV1-40*02	0/99									
	IGLV1-40*03	0/99									
CDR2-FR3	**IGKV1-13*01**	NA	ASSLESGVPSRF			✓	✓	47,5	\	37,8	33,5
	**IGKV1-13*02**	NA									
	IGKV1-5*01	24/99									
	IGKV1-5*02	0/99									
	**IGKV1-5*03**	93/99									
	IGKV1-5*04	NA									
	IGKV1-5*05	NA									
	**IGKV1D-13*01**	NA									
	**IGKV1D-13*02**	NA									
CDR2-FR3	**IGKV4-1*01**	96/99	ASTRESGVPDRF				✓	26,0	\	\	28,1
	IGKV4-1*02	NA									

FR3-CDR3	**IGHV7-4-1*05/IGHJ6*04**	0/99 + 1/99	ATLCSAEVCQCAEGK		✓		✓	✓	108,5/63,0	108,5	108,7/67,6	114,7/56,2
FR3-CDR3	**IGHV7-4-1*05/IGHJ6*04**	0/99 + 1/99	CSAEVCQCAEGK		✓	✓	✓		76,4	78,3	78,7	82,0
CDR2-FR3	**IGKV1-33*01**	NA	DASNLETGVPSRF					✓	\	\	57,4	49,6
	**IGKV1D-33*01**	NA										
CDR2-FR3	**IGLV3-21*02**	67/99	DDSDRPSGIPERF			✓	✓		18,8/26,6	30,8	22,8	\
	IGLV3-21*03	27/99										
CDR2-FR3	**IGLV3-21*02**	67/99	DDTDRPSGIPERF		✓				37,8	28,2	\	\
	IGLV3-21*03	27/99										
CDR2-FR3	**IGLV3-21*02**	67/99	DNSDRPSGIPERF					✓	\	\	\	27,4
	IGLV3-21*03	27/99										
CDR2-FR3	**IGLV3-27*01**	98/99	DSERPSGIPERF				✓	✓	26,5/21,0	16,4	28,1	31,3
CDR2-FR3	**IGLV3-27*01**	98/99	DTERPSGIPERF		✓			✓	31,1	28,0	31,3	31,5
CDR2-FR3	**IGLV2-14*04**	61/99	DVSNRPSGVSNRF			✓			22,4/22,2	40,5	16,3	8,1
CDR2-FR3	**IGLV1-44*01**	99/99	ENNQRPSGVPDRF		✓	✓		✓	26,3	27,7	\	27,8
	IGLV1-44*02	NA										
	**IGLV1-47*01**	94/99										
	IGLV1-47*02	22/99										
	IGLV1-47*03	0/99										
	**IGLV1-51*02**	25/99										
	IGLV6-57*01	43/99										
	**IGLV6-57*02**	68/99										
	IGLV6-57*03	25/99										
	IGLV6-57*04	NA										
CDR2-FR3	IGLV6-57*01	43/99	ENNQRPSGVPDRFSGSIDSSSNSASL		✓	✓		✓	36,6	35,9	22,6	21,8
	**IGLV6-57*02**	68/99										
	IGLV6-57*03	25/99										
	IGLV6-57*04	NA										
CDR2-FR3	**IGLV2-14*01**	64/99	EVSNRPSGISNRF			✓			\	23,2	22,6	22,9
CDR2-FR3	**IGLV2-14*01**	64/99	EVTNRPSGVSNRF			✓			28,0	24,0/25,6	31,2	17,8
CDR2-FR3	**IGKV3-20*01**	99/99	GASNRATGIPDRF					✓	\	\	\	21,4

CDR2-FR3	**IGKV3-20*01**	99/99	GASSRATGIPDRF		✓		✓	✓	26,0	19,4/21,7	26,3	30,2
CDR2-FR3	**IGKV3-15*01**	98/99	GASTRATGIPARF			✓		✓	26,8/25,8	25,7/23,4	31,9	26,8
	**IGKV3D-15*01**	99/99										
	IGKV3D-15*02	0/99										
FR2-CDR2-FR3	**IGHV4-34*13**	0/99	GLEWIGEINHSGSTNY				✓		50,2	36,2	47,6	42,5
FR2-CDR2-FR3	**IGHV4-34*13**	0/99	GLEWIGEINHSGSTNYNPSLK		✓		✓	✓	32,8	7,6	25,0	29,6
FR2-CDR2-FR3	**IGHV3-13*01**	77/99	GLEWVSAIGTAGDTYYPGSVK		✓	✓			87,5	112,5	\	\
	IGHV3-13*03	0/99										
	IGHV3-13*04	22/99										
FR2-CDR2	**IGHV3-53*01**	70/99	GLQWVSVIY		✓		✓	✓	34,2	32,6	35,4	24,6
	IGHV3-53*02	54/99										
	IGHV3-53*03	0/99										
	IGHV3-53*04	34/99										
	IGHV3-53*05	0/99										
	**IGHV3-66*01**	43/99										
	IGHV3-66*02	29/99										
	IGHV3-66*03	0/99										
	IGHV3-66*04	0/99										
	**IGHV3-23*03**	0/99										
	**IGHV3-NL1*01**	94/99										
CDR2-FR3	**IGLV1-40*01**	99/99	GNSNRPSGVPDRFSGSK			✓			35,3	29,8	\	\
	IGLV1-40*02	0/99										
	IGLV1-40*03	0/99										
FR1-CDR1	**IGLV3-19*01**	99/99	GQTVRITCQGDSL			✓			\	47,4	\	\
	IGLV3-19*02	1/99										
FR2-CDR2-FR3	**IGHV4-34*13**	0/99	IGEINHSGSTNY				✓		42,4	\	33,7	38,8
FR2-CDR2-FR3	**IGKV1-33*01**	NA	IYDASNLETGVPSRF				✓		\	101,4	116,0/39,7	125,7
	**IGKV1D-33*01**	NA										
FR2-CDR2-FR3	**IGKV3-11*01**	99/99	IYDASNRATGIPARF			✓	✓	✓	21,5/25,0	23,0	33,4	29,5
	IGKV3-11*02	0/99										

	**IGKV3D-11*01**	80/99										
	IGKV3D-11*02	58/99										
	IGKV3D-11*03	0/99										
CDR2-FR3	**IGHV5-51*05**	0/99	IYPGDSDTRY				✓		\	\	35,9	\
CDR2	**IGHV2-5*03**	NA	IYWDDDK					✓	\	39,4	39,2	39,2
FR2-CDR2	**IGHV2-70*02**	0/99	LALIDWDDDK			✓			57,6/53,2	57,5	53,7	\
	**IGHV2-70*07**	0/99										
FR2-CDR2	**IGHV2-70*05**	0/99	LARIDWDDDK					✓	29,6/55,6	27,4	\	30,6
FR3-CDR3	**IGHV7-4-1*05/IGHJ6*04**	0/99 + 1/99	LATLCSAEVCQCAEGK					✓	\	\	\	60,2
CDR2-FR3	**IGKV2-28*01**	NA	LGSNRASGVPDRF					✓	\	13,5	29,9	29,4
	**IGKV2D-28*01**	NA										
FR2-CDR2-FR3	**IGKV1-33*01**	NA	LLIYDASNLETGVPSRF			✓	✓	✓	73,1/101,4	139,5	125,8/52,0	125,7/52,6
	**IGKV1D-33*01**	NA										
FR2-CDR2-FR3	**IGKV1-33*01**	NA	LLIYDASNLQTGVPSRF		✓				139,9	\	\	\
	**IGKV1D-33*01**	NA										
FR2-CDR2-FR3	**IGLV1-51*01**	98/99	LLIYDNNK		✓			✓	53,1	43,1/47,8	47,3/47,5	47,6
FR2-CDR2-FR3	**IGHV1-69*03**	0/99	MGGIIPIFATPNY		✓			✓	22,9	\	\	21,4
FR2-CDR2-FR3	**IGHV1-69*03**	0/99	MGGIIPIFGTANY		✓	✓	✓	✓	47,0	44,9	26,8	22,4
FR2-CDR2-FR3	**IGHV5-51*05**	0/99	MGIIYPGDSDTRY			✓			78,3/78,3	78,2	45,2	\
FR2-CDR2	IGHV5-10-1*01	22/99	MGRIDPSDSY				✓		\	\	39,0	\
	IGHV5-10-1*02	0/99										
	**IGHV5-10-1*03**	57/99										
	IGHV5-10-1*04	0/99										
FR2-CDR2-FR3	IGHV1-69*02	13/99	MGRIIPILGIANY		✓	✓	✓	✓	56,4	51,0	54,4	50,2/29,1
	**IGHV1-69*04**	27/99										
	IGHV1-69*09	8/99										
CDR2-FR3	**IGLV1-44*01**	99/99	NNNQRPSGVPDRF			✓			27,6	25,9/24,5	26,0	3,7
	IGLV1-44*02	NA										
	**IGLV1-47*02**	22/99										
CDR2-FR3	**IGLV1-44*01**	99/99	NNNQRPSGVPDRFSGSK			✓			26,6	27,2	\	\

	IGLV1-44*02	NA										
	**IGLV1-47*02**	22/99										
CDR2-FR3	IGLV3-21*01	0/99	NSDRPSGIPERF			✓	✓	✓	\	35,0	38,3	38,3
	**IGLV3-21*02**	67/99										
	IGLV3-21*03	27/99										
	IGLV3-21*04	56/99										
CDR2-FR3	**IGHV3-9*01**	78/99	NSGSIGYADSVK			✓		✓	\	62,1	\	58,7
	IGHV3-9*02	0/99										
	IGHV3-9*03	2/99										
	IGHV3-9*04	NA										
FR2-CDR2	**IGLV3-27*01**	98/99	PGQAPVLVIYK					✓	\	\	\	58,7
FR2-CDR2-FR3	**IGLV3-21*02**	67/99	PGQAPVLVVFDNSDRPSGIPERF		✓				35,1	41,6	\	15,0
	IGLV3-21*03	27/99										
FR2-CDR2-FR3	**IGLV3-21*02**	67/99	PGQAPVLVVYDDSDRPSGIPERF			✓	✓	✓	55,9/48,6/40,0	52,0	63,3	59,3
	IGLV3-21*03	27/99										
FR2-CDR2-FR3	**IGLV3-21*02**	67/99	PGQAPVLVVYDDTDRPSGIPERF		✓	✓			48,0	51,6	49,7	\
	IGLV3-21*03	27/99										
FR2-CDR2-FR3	**IGLV3-1*01**	99/99	PGQSPVLVIYQDSK			✓			69,4	60,6	\	\
FR2-CDR2-FR3	**IGLV6-57*01**	43/99	QQRPGSSPTTVIYENNQRPSGVPDRF					✓	\	\	\	22,3
FR1-CDR1	> 100 possible IGHV3	NA	RLSCAASGF		✓	✓	✓	✓	36,8	36,8	37,0	34,8
FR1-CDR1	**IGKV1-33*01**	NA	SASVGDRVTITCQASQDISNY		✓	✓			90,4	52,4	\	\
	**IGKV1D-33*01**	NA										
FR1-CDR1	**IGKV3-20*01**	99/99	SCRASQSVSNTY				✓	✓	\	18,5	20,1	20,4
	IGKV3-20*02	0/99										
	IGKV3-7*01	2/99										
	IGKV3-7*02	1/99										
	IGKV3-7*03	0/99										
	**IGKV3-7*04**	92/99										
	**IGKV3D-20*02**	0/99										
	**IGKV3D-7*01**	62/99										

FR1-CDR1-FR2	**IGKV3-15*01**	98/99	SCRASQSVSSNLAW				✓		39,6	\	29,9	\
	**IGKV3D-15*01**	99/99										
	IGKV3D-15*02	0/99										
	IGKV3D-15*03	20/99										
FR1-CDR1	**IGKV3-20*02**	0/99	SCRASQSVSSSY		✓	✓	✓	✓	54,1	62,3	74,6	39,9
FR1-CDR1	**IGKV3-11*01**	99/99	SCRASQSVSSY		✓		✓		42,4	42,0	49,9	\
	IGKV3-11*02	0/99										
	**IGKV3D-11*02**	58/99										
CDR2-FR3	**IGHV4-34*13**	0/99	SGSTNYNPSLK			✓		✓	80,8	83,1	\	49,4
	**IGHV4-59*09**	0/99										
CDR2-FR3	**IGLV1-44*01**	99/99	SNNQRPSGVPDRF			✓			22,0	23,8	\	17,3
	IGLV1-44*02	NA										
	**IGLV1-47*02**	22/99										
CDR2-FR3	**IGLV1-44*01**	99/99	SNNQRPSGVPDRFSGSK			✓			37,2	43,4	\	\
	IGLV1-44*02	NA										
	**IGLV1-47*02**	22/99										
FR1-CDR1	**IGLV3-9*01**	0/99	TARITCGGNNIGSK		✓	✓	✓	✓	80,8/63,3	69,4	65,4	70,4
CDR1-FR2	**IGHV3-43*02**	11/99	TFDDYAMHW					✓	47,6/47,0	40,9/48,0	48,0/36,9	44,9
	IGHV3-43D*03	19/99										
	**IGHV3-43D*04**	22/99										
	**IGHV3-9*01**	78/99										
	IGHV3-9*03	2/99										
	IGHV3-9*04	NA										
FR2-CDR2	IGHV3-30*01	11/99	VAVISYDGSNK		✓	✓			56,6	35,1	\	13,9
	IGHV3-30*03	5/99										
	IGHV3-30*04	18/99										
	IGHV3-30*05	0/99										
	IGHV3-30*06	0/99										
	IGHV3-30*07	0/99										
	IGHV3-30*08	0/99										

	IGHV3-30*09	0/99										
	IGHV3-30*10	0/99										
	IGHV3-30*11	0/99										
	IGHV3-30*12	0/99										
	IGHV3-30*13	0/99										
	IGHV3-30*14	0/99										
	IGHV3-30*15	0/99										
	IGHV3-30*16	0/99										
	IGHV3-30*17	0/99										
	**IGHV3-30*18**	93/99										
	IGHV3-30*19	0/99										
	IGHV3-30*20	NA										
	**IGHV3-30-3*01**	70/99										
	IGHV3-30-3*02	0/99										
	IGHV3-30-3*03	NA										
	**IGHV3-30-5*01**	NA										
	**IGHV3-33*05**	0/99										
FR2-CDR2-FR3	**IGHV1-69*03**	0/99	VRQAPGQGLEWMGGFIPAFGTPVY				✓		\	\	20,8	\
FR2-CDR2-FR3	**IGHV1-69*03**	0/99	VRQAPGQGLEWMGGIIPIFGTANY		✓				40,1	32,2	\	\
FR2-CDR2-FR3	**IGHV3-13*01**	77/99	VSAIGTAGDTYYPGSVK		✓	✓	✓		70,8	71,5	56,3	\
	IGHV3-13*03	0/99										
	IGHV3-13*04	22/99										
FR2-CDR2-FR3	**IGHV3-74*01**	99/99	VSRINSDGSSTSY			✓	✓		48,9	32,1	60,0	\
	IGHV3-74*02	0/99										
CDR2-FR3	IGLV3-21*01	0/99	YDSDRPSGIPERF				✓		31,3	\	30,3	\
	**IGLV3-21*04**	56/99										
CDR2-FR3	IGLV3-21*01	0/99	YNSDRPSGIPERF			✓			\	20,9	\	18,2
	**IGLV3-21*04**	56/99										
FR3-CDR3	**IGKV5-2*01**	93/99	YSFCTDHTVL			✓			\	20,4	\	26,7
FR2-CDR2-FR3	**IGLV6-57*02**	68/99	YQQRPGSAPTTVIYENNQRPSGVPDRF					✓	\	\	\	38,5

	IGLV6-57*03	25/99										
	IGLV6-57*04	NA										
FR2-CDR2-FR3	**IGLV6-57*01**	43/99	YQQRPGSSPTTVIYENNQRPSGVPDRF					✓	\	\	\	23,7

Peptides that were higher before vaccination - *De novo* sequencing
(no discriminatory peptides were identified using an Uniprot *Homo sapiens* database search)

					PLS-DA Discriminatory peptides				PEAKS *de novo* Score (%)			
Region	Subject_ID	Allelic frequency observed in (2)	Sequence	*De novo*	Exp. 1	Exp. 2	Exp. 3	Exp. 4	Exp. 1	Exp. 2	Exp. 3	Exp. 4
FR3-CDR3	**IGKV2-28*01**	NA	LCMAALK	✓				✓	93	96/93	\	91
	**IGKV2D-28*01**	99/99										
FR2-CDR2-FR3	**IGKV1-33*01**	NA	LYNASNLETGVPARY	✓		✓			89	89	\	\
	**IGKV1D-33*01**	NA										
FR2-CDR2-FR3	**IGKV1-33*01**	NA	LYNVSNLETGVPSRF	✓		✓			98	98	\	\
	**IGKV1D-33*01**	NA										
CDR2-FR3	**IGHV3-11*01**	78/99	NGATLNYADSVK	✓		✓			97	97	\	\
	IGHV3-11*04	2/99										
	**IGHV3-21*07**	NA										
	**IGHV3-48*03**	34/99										
FR1-CDR1	**IGLV1-62*01**	0/99	SGTLACTGSLVNNTTGFVY	✓	✓				84	\	\	\
