# Supplementary figures and images for: Complementarity determining regions in SARS-CoV-2 hybrid immunity
Source: Front Immunol. 2023 Feb 21;14:1050037. doi: 10.3389/fimmu.2023.1050037 (PMC9990870; doi:10.3389/fimmu.2023.1050037)

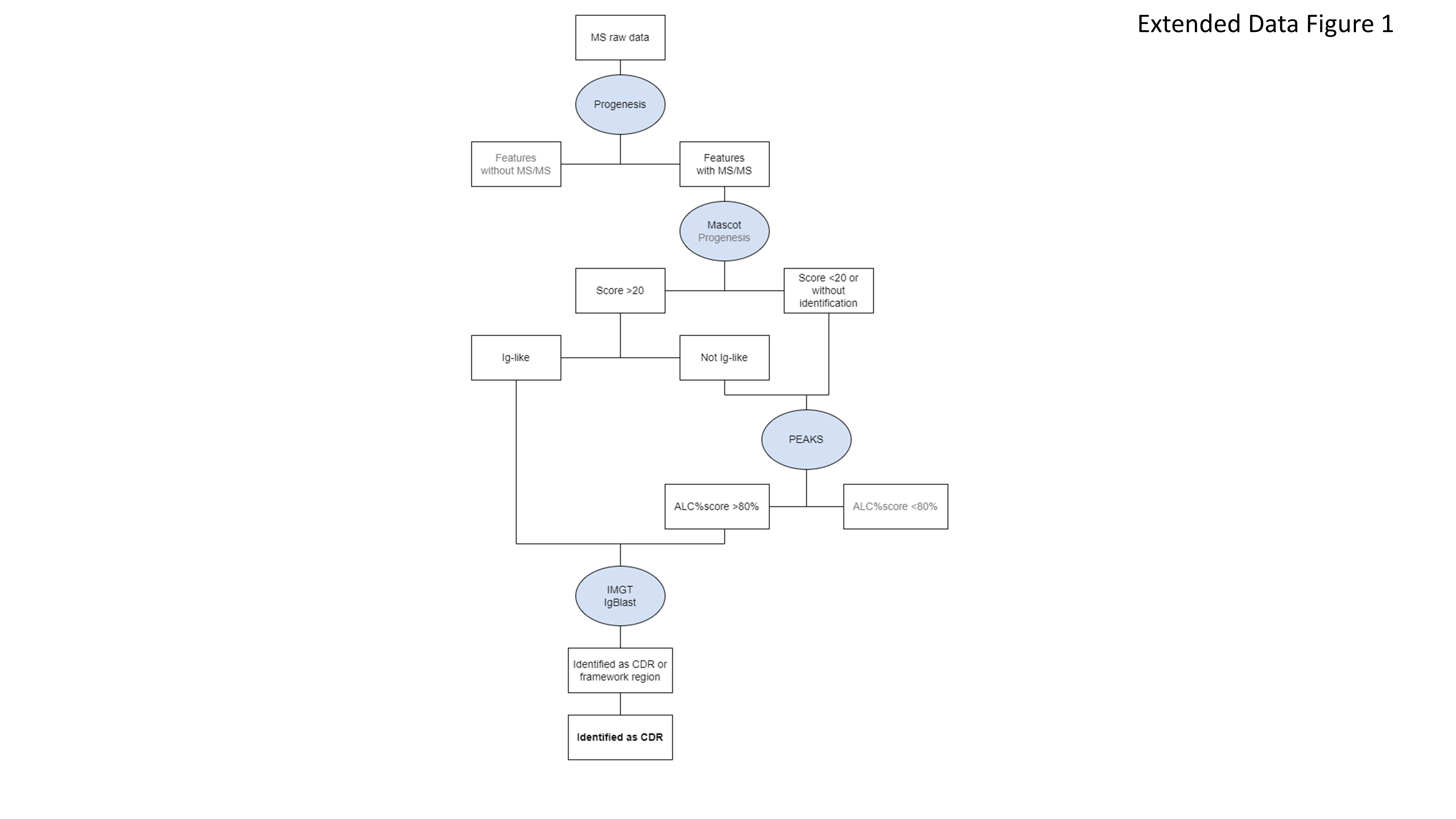

Supplement: Supplementary file 2 [file Image_1.jpg]

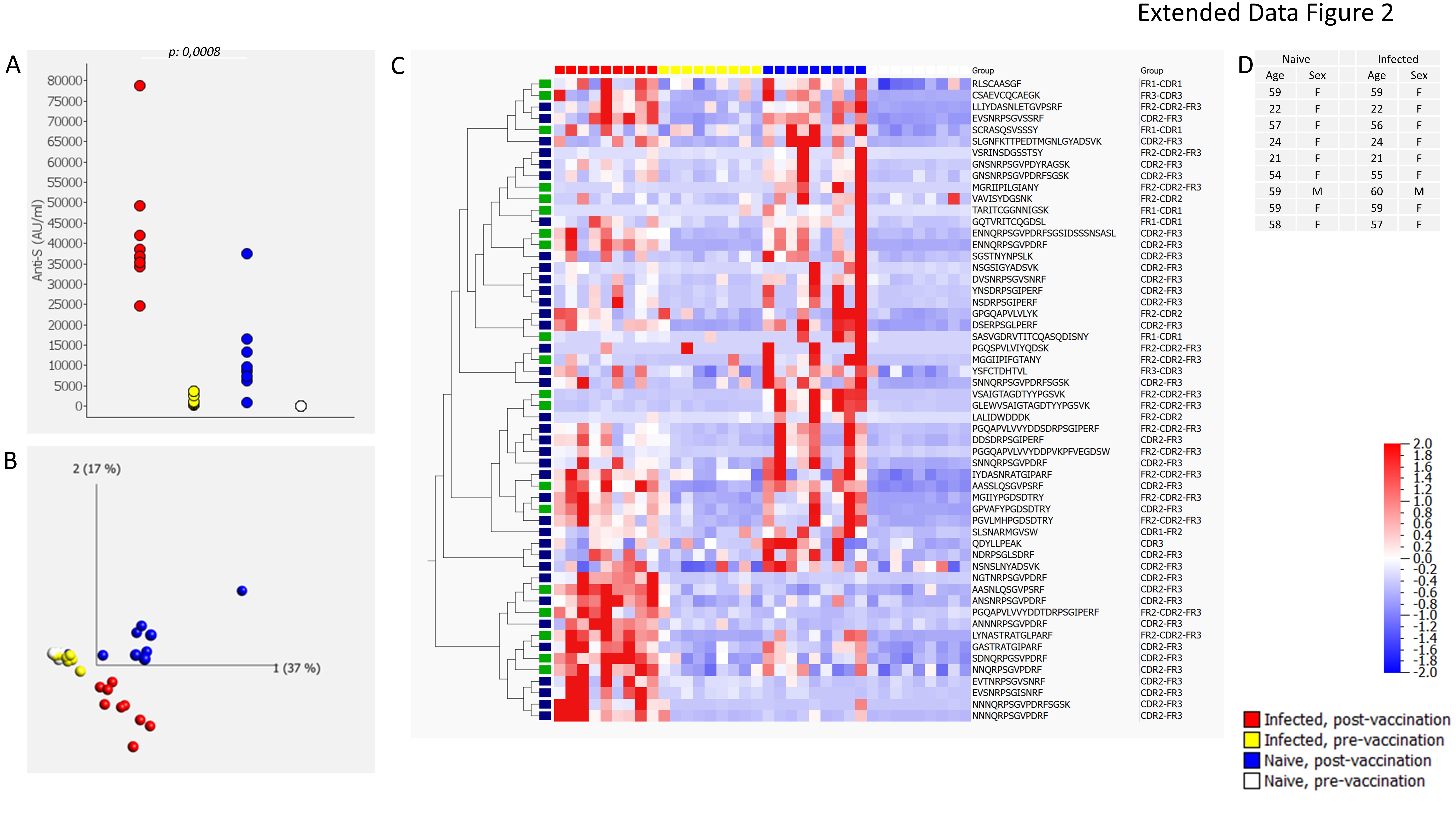

Supplement: Supplementary file 3 [file Image_2.jpg]

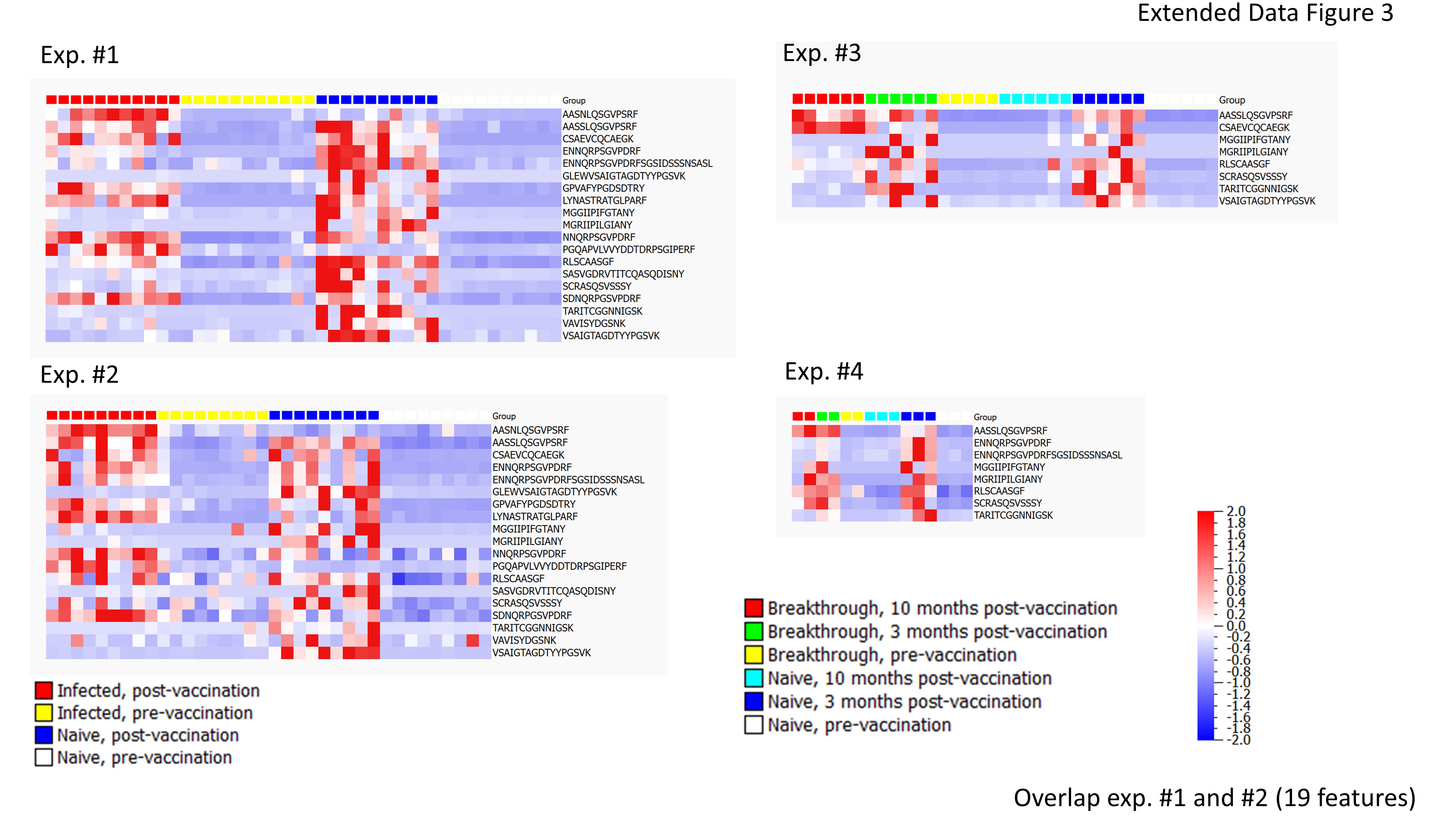

Supplement: Supplementary file 4 [file Image_3.jpg]

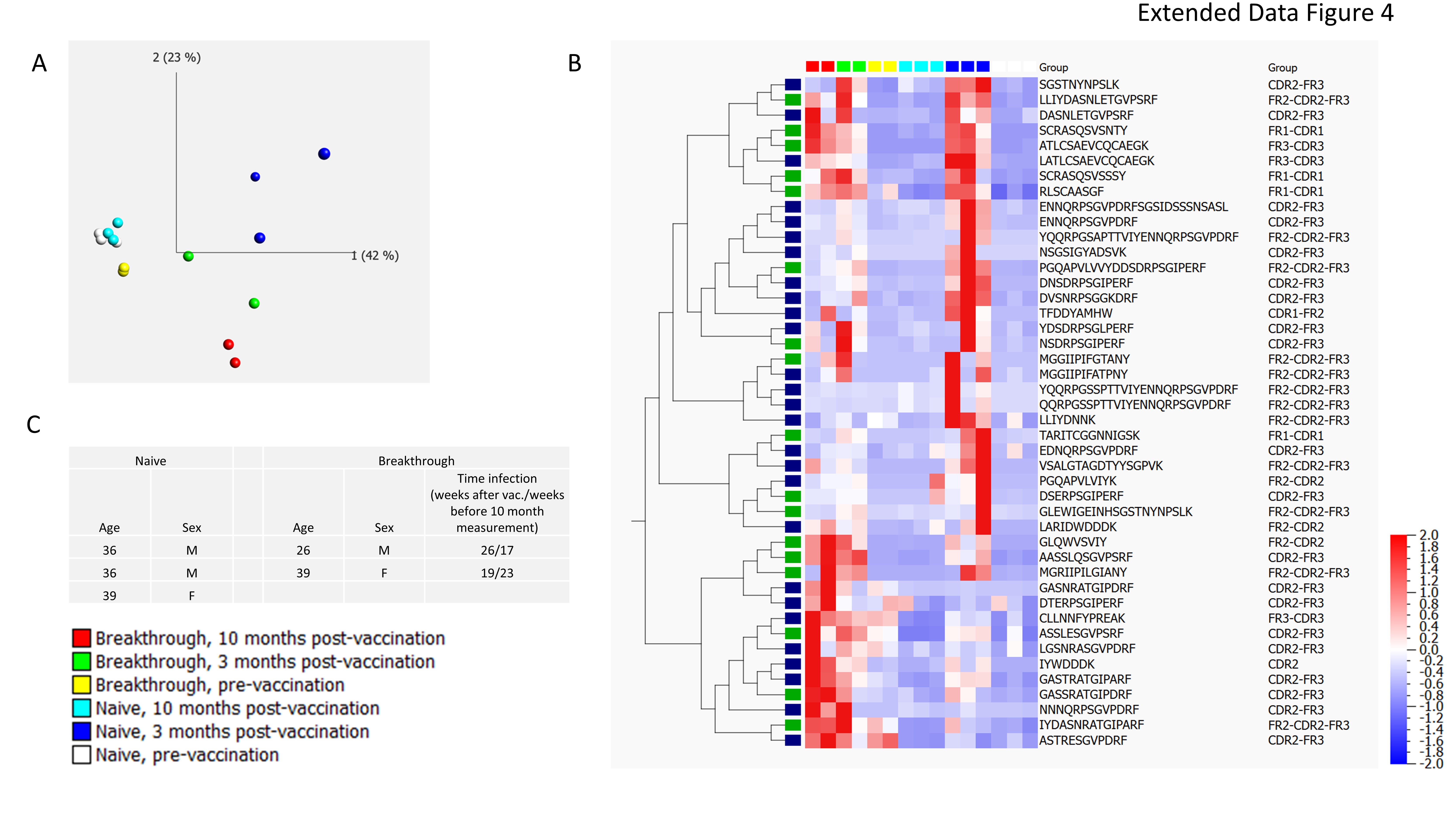

Supplement: Supplementary file 5 [file Image_4.jpg]

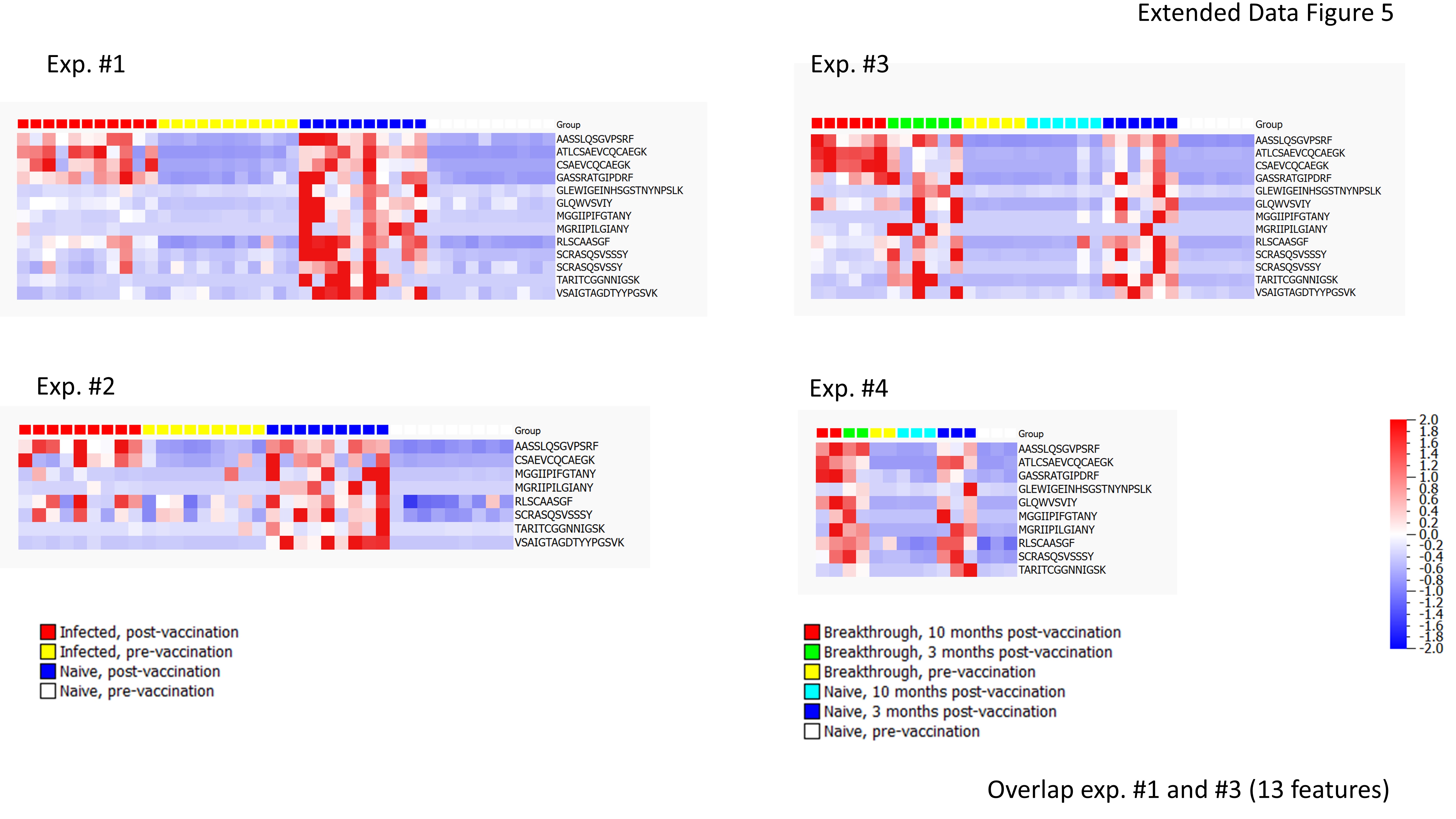

Supplement: Supplementary file 6 [file Image_5.jpg]
